# Self-perception evolution among university student TikTok users: evidence from China

**DOI:** 10.3389/fpsyg.2023.1217014

**Published:** 2024-02-19

**Authors:** Jinsheng (Jason) Zhu, Yan Ma, Guoen Xia, Sabariah Mohamed Salle, Hongye Huang, Shahrul Nazmi Sannusi

**Affiliations:** ^1^Faculty of Social Science and Humanities, Centre for Research in Media and Communication, Universiti Kebangsaan Malaysia, Selangor, Malaysia; ^2^Belt and Road International School, Guilin Tourism University, Guilin, China; ^3^School of Journalism and Communication, Guangxi University of Finance and Economics, Nanning, China; ^4^School of Business Administration, Guangxi University of Finance and Economics, Nanning, China; ^5^School of Journalism and Communication, Nanning Normal University, Nanning, China

**Keywords:** short video, value formation, university students, self-perception, TikTok (Douyin in China)

## Abstract

The effects of short movies on social media platforms are gaining worldwide popularity and are now attracting global academic attention. Employing self-perception theory and qualitative research methodology, the study examines the influence of short video applications (TikTok) on app-user engagement and evaluates the self-perceived cognitive psychological understanding of Chinese university students. The findings show that identity, attitude change, emotional perception, and civic engagement are the most influential aspects of Chinese youths’ self-perceptions. Furthermore, the positive and negative correlated components influence the distribution of short video values. Such tactical use of personality construction contributes to the present psychological research of Chinese university students.

## Introduction

The viewership of short videos is growing in popularity all around the world (Wang, 2020). However, there are heated discussions in the research literature concerning its relevance and prospective impacts, notably its implications on the personality evaluations of adolescents (Jarman et al., 2021), social wellbeing (Best et al., 2014) as well as isolation and loneliness (Leigh-Hunt et al., 2017). People can learn from their own thoughts, sentiments, and other emotional reactions in part by inferring them from their observable behaviour or the world of uncertainty in which such behaviour occurs (FeldmanHall and Shenhav, 2019). As a kind of social perception, self-perception is a person’s subjective perception of oneself, the psychological basis of behaviour, and a complex psychological process (Turner and Oakes, 1986). Perception, experience, needs, and motivation all impact a person’s self-evaluation and self-judgment, which are accomplished by self-control, self-evaluation, and other self-learning processes (Fauzi and Widjajanti, 2018). These are the most important indicators of a person’s psychological health, and ultimately, overall psychological well-being (Houben et al., 2015). TikTok has gained significant popularity and broad adoption among young people and Generation Z, making it one of the most popular applications (Hase et al., 2023; Schellewald, 2023). Regarding this matter, the research not only focuses primarily on TikTok as the major platform to be analysed in this paper. It is crucial to write a document that thoroughly explains the platform, emphasizing its capabilities and affordances (Cervi, 2021), its cultural context in-between (De Leyn et al., 2022), and its usage by Generation Zers (Stahl and Literat, 2023). It is crucial to recognize and examine the existing extensive body of literature on this subject in order to provide a solid foundation for the research. Therefore, it is also essential to explore the self-perception aspect while delving into the study of TikTok via an academic perspective.

It is becoming increasingly important for academics to start investigating how applications for creating short videos, including TikTok, are affecting Chinese youths’ cultural practices and their psychological patterns of interpersonal perceptions. This study investigates how the rise of the video-sharing platform TikTok is impacting traditional Chinese cultural traditions and psychological modes changing. The present study intends to investigate the following research questions: (1) How do elements within short video content contribute to biases in the transmission of values, particularly in relation to self-perception? (2) What are the primary factors that influence distortions in self-perception resulting from the communication of values through short videos? (3) To what extent does the prevalence of TikTok influence the self-concept and daily practices of Chinese university students, encompassing their entertainment preferences, behavioural patterns, and evolving cultural dynamics. To further investigate these research questions, a combination of quantitative and qualitative research methods was applied to the study of the most widely prevalent short videos on TikTok among Chinese university students from the years 2012 through 2022. The goal of this research would be to determine the extent to which the popularity of TikTok has an impact on the participants’ conceptions of themselves as individuals. The outcomes of this study show how short video apps, which are an essential component of the lifestyles of Chinese university students, affect their day-to-day practises, including their amusement preferences, self-perception, behavioural patterns, and younger cultures.

The following sections are set as follows: First, the authors started to summarize related literature to the topics, including the issues of self-perception of symbolic interaction, attitude, psychological emotion, and social comparison. Second, the next section introduces the research methodology, which includes a semi-structured interview and a grounded theory approach. Research findings would be displayed in the following section, together with the discussion and conclusion sections. The present research contributes to a deeper understanding of the philosophic, theoretical, and methodological interpretations of the theory of self-perceptions about TikTok users, particularly teenagers and university students.

## Literature review

### Self-perception of symbolic interaction

Sociability, information sharing, communication, and self-perception are inescapable aspects of everyday lives that need symbolic interaction (Daniels, 2021). Self-perception is a fundamental component of symbolic interaction since one must participate in symbolic contact in order to see the self, others, and the symbolic meanings they hold (Stets and Burke, 2003). Individuals are able to communicate more actively with others on social networks to express themselves and form connections (Caton and Chapman, 2016). Consequently, symbolic interaction as a sort of behaviour is therefore influenced, resulting in an influence on each of us in society.

Symbolic interaction as a form of behaviour is often thought of as people interacting with each other, while self-perception is relatively uncommon. When symbols are expressed in different ways, they are perceived differently and therefore affect the perception of others. In particular, Mckenny (2012) suggests that social identification with the benefit stakeholder group and competence with the group’s need both increase the possibility of stumbling onto and making use of an opportunity for social entrepreneurship. In this regard, Wallace et al. (2012) conducted research to investigate whether or not adolescent’s locus of control influenced the relationship between self-perception factors and aggressiveness, which mainly consists of (1) user interaction was related to self-perception; (2) behaviour was mainly influenced by personal interests.

Consistent with previous research, it has been shown that symbolic interaction may lead to self-perception, which can impact others and transform oneself, given that people see different symbols in different ways and therefore construct self-perception (de Vries and Kühne, 2015). In the preceding part of the introduction, we expounded on this subject in connection to pertinent research in order to give a theoretical foundation and research gap for future study. The part that follows will continue to build on and explore self-perception within the context of relevant studies.

### Self-perception and social networking

People communicate symbolically with one another in social networks (Aakhus et al., 2012; Kane, 2012), which is more of a result of symbolic expression and engagement with other people than of a feeling or a sensation. People have diverse ways of seeing and depicting other people depending on the situation in which they find themselves; as a result, individuals build their own self-perceptions and influence others via symbolic relationships. In terms of social interaction, when we feel that others recognize us, we will interact with each other actively, which is consistent with our cognitive level. In the meantime, we will have greater psychological demands to gain the recognition and support of others (Wong, 2022), and when we feel that others recognize us, we will make greater efforts to satisfy others, and we will communicate with them symbolically more effectively. When we are the recipients of compliments and appreciation from others, we are motivated to do more to win their approval and are better able to connect with them on a metaphorical level, which ultimately results in the creation of more shared ideals and the strengthening of ourselves. A positive element of social networks is that they enable us to transmit our ideas and emotions to others symbolically (Seng et al., 2018). This makes it simpler for people to identify themselves in order to establish contacts and communicate. When taken together, the above arguments demonstrate to the utmost extent that mental activity and symbolic connection both affect how we see ourselves and our feelings, while simultaneously being effectively impacted and inspired (Janicke et al., 2018). Four significant types of symbolic interaction are outlined below: interaction, symbolic cognitive activity, symbolic expression, and self-perception.

**Figure 1:**
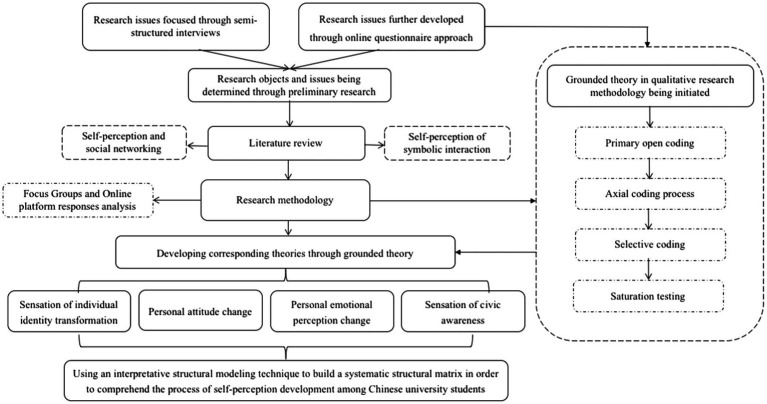
Conceptual research framework for understanding the self-perception evolution among TikTok users in Chinese universities.

Self-perception is an aspect of consciousness that is an organic component of the interaction and communication that makes up civilization (Keromnes et al., 2019). This indicates that, from the standpoint of human-computer interaction, we could investigate the dissemination of concepts via the use of short videos (Szolin et al., 2022). Such an understanding, which integrates human self-perception with the social environment, transcends the limitations of human-computer interaction. It also incorporates social engagement with other individuals, reflection on behaviour, and the investigation of the alteration of human self-perception in the cultural ecology of short videos from the standpoint of symbolic interaction (Charmaz et al., 2019; Meltzer et al., 2020). This strategy may also be accomplished by integrating human exposure and reactions to short videos, a radical alternative for action rather than the actor that harkens back to symbolic interactionism (Szabla and Blommaert, 2020; Namisango et al., 2022). Through a summary of the available literature, this paper synthesizes the associated research viewpoints, methodologies, and contents, so producing the research framework outlined below (see Figure 1).

## Mixed research methodologies

Similar to the research of Dearnley (2005) and Mcintosh and Morse (2015), the current paper used semi-unstructured interviews to identify key measures of self-perception, environmental perception and online behaviour.

### Adopting semi-structured interviews in qualitative research methodology

The semi-structured interview may be done without a predetermined standard questionnaire or research question form (Dearnley, 2005; McIntosh and Morse, 2015; Bahari et al., 2022). The interviewers are just given a basic direction for the subject of the interview, around which the authors and interviewees are allowed to converse (Rowley, 2012; Rutakumwa et al., 2020). As noted by Brönnimann (2022), the authors should be in favour to protect the situation from change and constantly modifying the structure of the questions based on the current context of the interview. During the course of the interview, the interviewers are flexible to change some directions when inspiration strikes or go further into a certain topic (Kvale, 2003; Morrison et al., 2019). In contrast to structured interviews, semi-structured interviews are frequently used to gain insight into complex facts that cannot be captured by fixed-procedure questionnaire surveys (Diefenbach, 2009; Brinkmann, 2014; Magaldi and Berler, 2020). This is particularly the case when it comes to essential aspects such as personal attitudes, values, and intentions that are difficult to summarize directly in standard questions (Foerstl et al., 2021). Thus, semi-structured interviews, which are used far more often to permit quantitative analysis of the information obtained from interviews, are adopted in the present study.

Numerous techniques for evaluating the self, perception, attitude, cognition, and emotion emerged with the advent of social cognitive psychology research, and numerous scientists developed scales for this purpose (Bandura, 2001; Barone et al., 2012). Due to the advantages of qualitative research (Alvarado and Waern, 2018; Yin et al., 2021), in this study, semi-structured interviews were utilized to determine the human-computer interaction aspects of short videos that impact individuals’ perceptive value. Based on the precedential research, the authors create a series of questions meant to elicit information about the research participants’ state of mind or the mental processes they have engaged in while watching the TikTok app, and the research participants respond to each open-end question based on how it resonates with their own subjective experience. The current study gives participants a standardized interview subject to facilitate free-flowing conversation (Dempsey et al., 2016). For the following part of the empirical study, the interviewees’ remarks collected from the semi-structured interviews were transcribed into texts and further analysed using content analysis to summarize the self-perceived components of the short video that influence value interaction.

### Adopting a grounded theory approach in qualitative research methodology

The grounded theory, which was established by Strauss and Glaser (Glaser and Strauss, 1964; Glaser et al., 1968), stresses the researcher’s capacity to engage completely with empirical data and to progressively generalize fundamental conceptualizations (Zahrai et al., 2022). The advantage of grounded theory research is that it takes the mystery out of qualitative research and moves beyond descriptive research into the realm of interpretive theoretical frameworks (Birks and Mills, 2011; Zhu et al., 2022). The qualitative material is fragmented, requiring the researcher to group it into corresponding themes, constantly comparing the commonalities that exist in all the qualitative material (Klykken, 2022). The gathered themes from the study were contrasted based on their substantial differences across classifications (Cabi, 2018; Choi et al., 2020). In the course of comparing, evaluating, integrating, and classifying qualitative data, and finally distilling novel theoretical concepts, this procedure is intended to provide a rational conceptual analysis (Jackson, 2015).

### Sampling

As a result, eight universities in Guangxi, including comprehensive postgraduate universities, normal undergraduate universities, finance and economics undergraduate universities, medicine undergraduate universities, and vocational colleges, were chosen to conduct interviews with students. Students from eight different universities, including both undergraduates and postgraduates at their respective institutions, participated in the interviews. Table 1 contains the basic information for 33 respondents, whose actual identities are substituted by code names.

**Table 1:** Demographic information about the student interviewees.

Pseudonym	Gender	Age	Education	Daily mobile phone screen time (Including usage of short video platforms such as TikTok)
ZS	Male	35	PhD	Above 3 h
XYW	Male	20	Undergraduate	Above 2 h
TJF	Female	19	Undergraduate	Above 4 h
YRY	Female	19	Undergraduate	Above 4 h
YF	Female	22	Junior university student	Above 4 h
ZYL	Female	22	Undergraduate	Above 3 h
LSY	Female	20	Junior university student	Above 3 h
YH	Male	29	PhD	Above 8 h
LSY	Female	21	Undergraduate	Above 2 h
LY	Male	24	Postgraduate	Above 3 h
HX	Female	19	Undergraduate	Above 4 h
HYM	Female	27	Postgraduates	Above 5 h
HHJ	Male	22	Undergraduates	Above 3 h
LMS	Male	26	Postgraduates	Above 8 h
CFY	Male	18	Undergraduates	Above 2 h
DHY	Male	20	Undergraduates	Above 2 h
ZSX	Male	19	Undergraduates	Above 6 h
HJJ	Male	38	PhD	Above 2 h
SZQ	Female	32	Postgraduates	Above 3 h
LSS	Female	29	Postgraduates	Above 3 h
CYJ	Male	19	Junior university student	Above 4 h
WYX	Male	28	Postgraduates	Above 3 h
LDY	Male	20	Undergraduates	Above 8 h
LJ	Male	28	PhD	Above 4 h
ZF	Female	20	Undergraduates	Above 6 h
ZY	Female	27	Postgraduates	Above 4 h
LS	Male	20	Undergraduates	Above 3 h
LWY	Male	19	Undergraduates	Above 6 h
XRH	Female	25	Postgraduates	Above 2 h
ZB	Male	27	PhD	Above 4 h
HH	Female	24	Postgraduates	Above 3 h
ZQ	Female	25	Postgraduates	Above 5 h
CS	Female	29	PhD	Above 4 h

The rightmost column in this table is labelled as “Daily mobile phone screen time (including usage of short video platforms such as TikTok)” to account for a mobile phone application that calculates daily and weekly screen time usage.

In the application of the grounded theory research method, it is essential to provide the applicable concept and theoretical framework for data analysis with the speculative idea, to treat the theoretical accomplishments of predecessors dialectically, and to consider the pertinent theories under specific conditions. Simultaneously, the authors’ argumentative reasoning and empirical knowledge then permeate the right application of the predecessors’ theories. The next step is to construct an adaptable, substantial, and reliable theory of inspiration based on empirical shreds of evidence.

### Interview process

An interview time is reserved with the interviewee for a face-to-face conversation. The open-ended questions of the interviews focused on the following four themes: (1) What perspectives were shown in the short TikTok videos? (2) What cognitive dissonances were conceivable? (3) What positive or negative emotions do they experience when exposed to short TikTok videos? (4) Does the process of observing include introspective rationality of self-perception? The respondents were simply instructed to describe their attitudes, emotions, and actions after viewing the short videos, and were encouraged to share their actual sentiments. Following the approaches of Sutton and Austin (2015), the data was then simplified and summarized in accordance with the field notes and voice recordings. The interview was limited to 30 min to reduce participants’ fatigue, which might lead to disengagement and discrepancies in the discussion. The interview was conducted to provide the interviewee with a positive atmosphere by asking encouraging questions and to create an environment comparable to a casual discussion for the interviewee so that the most genuine information could be acquired (Emans, 2019). Over the same interview process, data were collected on participants’ ambient cognitive bias and online behavioural bias during the short video exposure. After the interview, it was sufficient to organize the textual data received in the words into the relevant categories.

#### Open coding process

During the phase of the open coding process (Denzin and Lincoln, 1998; Brannen, 2004), the current research evaluated and analysed the initial responses of the student participants, as well as synthesised the themes and theories. Data analysis and coding are the foundation of grounded theory (Bate et al., 2019; Baek et al., 2021). Because qualitative resources are dispersed and fragmented, the authors must synthesize them into appropriate themes, continually analyse the commonalities across all qualitative materials, and evaluate the key distinctions within categories (see Tables 1, 2). In the process of comparing, analysing, merging, and classifying, qualitative materials are subjected to a credible category analysis, and fresh theoretical perspectives are ultimately retrieved (Konecki, 2019; White and Cooper, 2022). The main objective of this study is to establish the association between a macro theory and a micro key factor for the value transmission bias of short video by using the grounded theory research approach. The authors’ consideration of the case’s most influential aspects leads to the deductive hypothesis (Gilgun, 2019). In the use of the grounded theory research method, it is critical to regularly compare theories with empirical data, followed by a synthesis of the data’s and theories’ interconnections in order to extract relevant categories and their fundamental thematic attributes (Sarkar and Banerjee, 2023).

**Table 2:** Three-level theme division of self-perception.

First level	Second level	Third level
Self-perception	The sensation of individual identity transformation	Career perceptions
		Impression prediction
		Image differentiation
	Personal attitude change	Visual stimulation
		Utility satisfaction
	Personal emotional perception change	Real information
		Emotional outburst
		Emotional transformation
	The sensation of civic awareness	Entertainment satisfaction
		Freedom of choice
		The critical thinking

The interview provides evidence that self-perception may be broken down into three distinct levels of subject matter. On the basis of the facts presented here, we are able to come to the conclusion that there are four second-level themes the level of the first-level theme of self-perception. There are 11 third-level themes following the level of the second-level theme. Table 2 presents the detailed synopsis for the current perusal.

#### Axial coding process

In this process, this study has efficiently categorized the self-perception theories in the disciplines of psychology, communication, sociology, etc., and presented the preliminary applied theories ahead of time (Zhang Y. et al., 2020). The data gathered via the interview session were sorted, classified, and categorized accordingly (Conrad and Tucker, 2019). After a thorough analysis of the data in the class and genus, the data with comparable features were grouped and categorised into a single attribute, i.e., distinct attributes were separated under different classifications (Kendall, 1999; Vollstedt and Rezat, 2019). The distinctions between classes and characteristics should be compared continually and with deliberation. Finally, the preliminary descriptions of theories and conceptual genera are compared to assess their link. Considerations were given to whether the theory or the classification of genera may be altered. All of these will contribute to developing a scientific and credible theoretical framework for the next phase of empirical inquiry, as stated in Table 3.

**Table 3:** The open coding – generalized open coding – axial coding – selective coding process.

Opening coding (labelling) to initial statements from the interview participants	Opening coding (conceptualization)	Axis coding (categorization)	Selective encoding (core categorization)
Admire the doctor when watching the life-saving video. Some travel videos recommended routes, food trustworthy. We media will perform better and more professionally.	Positive career motivation Introduction to Occupational Environment	1. Career perceptions	The sensation of individual identity transformation
Dressing up, inserting yourself into movie characters, Posting on moments. Used all the smart image functions.	Feel ourselves Show ourselves	2. Impression prediction	
Watching videos and live streams of younger girls, making jokes, and making friends with strangers.	Virtual social interaction	3. Image differentiation	
Not used to reading too long text When watching those (video) snippets, time goes by in a flash.	Immersed in short videos Kill time	4. Visual stimulation	Personal attitude change
Retweeting short videos is a way to display a personal hashtag, I will retweet fitness, and healthcare content, and jokes to family groups.	Satisfaction of motivation Consumption of culture	5. Utility satisfaction	
I feel that the short videos pushed by TikTok are quite good at first, but after a while, they are always the same thing.	Experience of emotion Information cocoon room	6. Real information	Emotional perception change
Sometimes I hate the themes that TikTok recommends. For example, when the thief got out of jail, TikTok was full of him. I think the video of the beautiful girl in Chengdu looks false.	Conflict with a video theme Negative emotions after viewing	7. Emotional outburst	
I like to see young girls in TikTok, and I will paint a lot of gifts for them because I do not get to know so many girls in real life. If they do not feed back to me, I would be disappointed.	Negative emotional interaction Virtual social addiction	8. Emotional transformation	
Usually, TikTok sends me the content of comics. I like drawing and playing games, such as quadratic elements. I prefer watching beauties in TikTok and watching variety shows. The teacher recommended excellent media coverage, but I do not watch, I prefer entertainment shows.	Pursuit of the nonconformity Pursuit of entertainment	9. Entertainment Satisfaction	The sensation of civic awareness
The leisure and entertainment function of the short video platform does not affect one’s attention to current events. Although TikTok does recommend some news, it is very rare. It is mostly entertainment	Pay close attention to current political issues Prefer the theme of leisure and entertainment	10. Freedom of choice	
Video journalism is more attractive than in-depth text reporting; Watching videos is much more comfortable than reading those long reports I will go to see the freezing point or Pengpai news for deep reports	Ditch deep thinking	11. The critical thinking	

The decision to employ grounded theory as the research methodology in this study aimed to conduct a thorough analysis of theoretical loci, inductive thematic patterns, and data saturation (Saunders et al., 2018). This saturation approach streamlined our research process, from the initial theoretical conceptualization and data collection to thematic analysis and theoretical refinement (Charmaz and Thornberg, 2021). To confirm with the objective of data saturation, we did another run of semi-structured interviews to the interviewees. Consequently, the interview results indicate that there were no new themes emerged from the interviews, affirming the adequacy of theme extraction and the alignment of the study’s theoretical focus. Thus, the data saturation process indicated that there were no further samplings and interviews needed for the current study.

The four themes relating to university students’ self-perceptions are further developed after examining the important aspects that contribute to the student’s self-perception of the worth of short videos are extracted using grounded theory research. This section attempts, through empirical and quantitative analysis, to achieve accurate attribution of third-level themes and to discover the primary factors influencing the value perceptions of short videos to university students. On the basis of this categories framework, the self-perception theory model asserts that short video consumers undergo an identity transformation in response to stimuli and subsequently create a matching physiological output (Hsiao, 2017). Hsiao (2017) argues that the obsessive use of smartphone apps might negatively impact people’s psychological health and social relationships. Underneath the influence of these short videos, subscribers may display different manifestations of social networking exhaustion. Based on a such line of thinking, a total of 11 categories (see Table 3) among the three dimensions of self-perception of social media impacts produced from the above analysis according to the grounded theory. The frequencies of various coding elements reported by the viewers of the short video among Chinese university students varied from one another, as shown by the frequencies of the statements shown above.

## Research findings

### The sensation of personal identity reformation

According to the interviews conducted, exposure to a multitude of videos plays a pivotal role in shaping an individual’s sense of identity. In this research, various factors contribute to the sensation of personal identity transformation, including one’s perception of their anticipated future occupation or identity, the alignment of self-perception with external perceptions, and the comparative analysis of one’s real self with their virtual social interaction self-image. In alignment with studies conducted by Parry et al. (2020), the utilization of social media platforms like TikTok, through video content consumption, lays the foundational groundwork for shaping life principles at the level of self-perception. Personal attributes such as courtesy, tranquillity, appreciation for labour, integrity, and benevolence emerge as fundamental components in the construction of individual value systems. For instance, an interviewee shared the following perspective:

*I adore viewing videos on Tiktok that depict physicians saving lives and healing people in my everyday life. By watching these movies, I am also able to comprehend the challenges that the medical field we are studying will face in the future, while simultaneously gaining confidence and gaining insight into a future medical career. This should be the procedure for developing and distinguishing the representation of the professional path that we students identify with.* (LY is currently a graduate student at the institution, where he is majoring in medicine. During the interview, he offered a positive reaction to the consequences of app-viewing behaviour. The interview was conducted at some point in time prior to June 2022.)

The intensity of students’ identification with specific coding components impacts these metrics, consequently influencing the construction of a distinct self-identity through individual reflection (D’Angelo and Ryan, 2021). Students gain a profound understanding of the importance of mastering this professional skill through the social connections fostered by short video content. This process facilitates the establishment of one’s identity through self-presentation. It is posited that students delineate their self-presentation and formulate unique self-identities, driven by diverse motivations and objectives triggered by their consumption of short video content.

### Personal attitude change via watching the TikTok short videos

Yeshurun et al. (2021) propose that the default mode network is a dynamic and responsive sense-making network that combines current extrinsic information with pre-existing intrinsic knowledge to construct complex, context-dependent situational models. As shown in this study, these self-perception changes can occur as a direct or indirect outcome of the short video-viewing experience. The emotional resonance that people experience with the content symbols as a reaction to the visual stimulation of short movies is one of the topics that are present in the higher education section. In this research, HX believes she has a negative attitude and emotion for the short video clip in which she displayed the following statements.

*The initial push of the positive short video left me feeling good. The problem is that they remain the same items, for instance, the information about celebrities, rumours, and cosmetics. Over years of viewing these movies, it seems that the app makes it hard to determine how often they alter the themes that are pushed on me. It reminds me of the "information cocoon chamber," only there does not appear to be any way to escape it.* (As a 19-year-old university student, HX criticized and questioned the information-pushing mechanism of short-video social media platforms. The interview took place in June 2022.)

The extent to which watching short videos meets the individual’s particular wants and requirements for information, and the consequent changes in the individual’s attitudes about short video content as a result of these changes. Individuals acquire emotional experiences via media engagement. This is based on genuine information that we can rapidly and correctly collect, which is both knowledgeable and useful. Individuals prefer to invest a certain amount of confidence in media outlets that convey a feeling of informational veracity. For instance, the benefits of authoritative media in terms of communication, advice, influence, and legitimacy allow them to play an important role in value orientation. A poor example of duplicate information experience is the TikTok video pushing mechanism, which has been roundly criticized by university internet users.

### Emotional perception change

Yeshurun et al.’s (2021) study show that an individual’s effective perception and experience of changes in their own emotions during exposure to short videos. In this process, the themes influencing perception change include one’s own experience with the authenticity of information and the resultant rise or reduction in confidence in the source seen by university students. The emotional outbursts that occur in short-video-based virtual interaction. Therefore, the creation of positive or negative emotions in response to short video exposure influences the emotional perception of Chinese university students. Emotional normalcy in the cultural ecology of short-form video is a process of a connotation for people, and excellent self-awareness may assist university students in developing long-lasting, healthy personality qualities that contribute to the stability of social values. LS, one of the interview participants, has a negative view of the use of short videos to exhibit bloggers’ hashtags and an emotional aversion to some of the suggested content.

*When that (faking images and videos) happens, the videos that Tiktok messages to me may be rather frustrating. In particular, there are several shots of a burglar who took the battery off of an electric bike and displayed them online. Additionally, the videos and images of females in Chengdu city are mostly fabricated and illusioned without a solid foundation.* (LS is a second-year student at one of the institutions that participated in this research. He is majoring in hotel management. The interview was carried out in the location where he was interning and working in July of 2022.)

The comments to this interview reveal that, based on a greater grasp of university students’ thought processes, contemporary Chinese university students are seeing and analysing TikTok videos on a deeper level. Students engage in a four-part cycle of planning, implementing, observing, and commenting on the process of video viewing in an effort to develop reasonable mechanisms for applying their off-campus learning. In this study work, the authors base their pedagogical and instructional activities on a consideration of this occurrence and the subsequent stage.

### The sensation of civic awareness

During their exposure to short videos, university students’ concern for information in the public domain either increased or decreased. The higher education themes include changes in the amount of energy devoted to social and public affairs as a result of the influence of entertainment messages and the cost of reflection required to comprehend and assimilate short-form content. The components of civic awareness suggest that, in the context of short-form video, there must be variations in persons’ attention to national public issues, which are directly related to the construction of one’s basic values at the national and social levels. As a typical member of contemporary university students, TJF shows a certain lack of civic consciousness.

*TikTok only gives me humorous material. In my leisure time, I like sketching portraits, particularly of gorgeous, featured females. I like playing video games as well. TikTok allows me to view them all. The teacher also suggested that we read more excellent media reports and public accounts, such as People's Daily and China Youth Daily, but I didn't read them much. I did not see any current political news. However, as for me, I hardly follow current political events.* (TJF is a first-year undergraduate student attending one of the institutions that were researched for this article. Through her consumption of Tictok videos, she communicated her disinterest in both the recent news and her participation in public life. The interview was scheduled for August of 2022 and was taking place at the institution.)

For Chinese university students, new media and modern news education have evolved simultaneously. The fast expansion and broad usage of new media have resulted in dramatic changes in the way information is distributed and people connect (Zhuravskaya et al., 2020). Compared to conventional media, new media is relatively developing in a new formation (Etter et al., 2019). The new media refers to both the interactive peer-to-peer state of communication and the altered ecology of information delivery. New media communication is distinguished from conventional media by the immediacy of the communication state, the interactivity of the communication topic, and the breadth of the communication scope. In terms of future development patterns, new media will undoubtedly thrive with the significant developments in the Internet and digital technologies. The steady demise of the old media business and the trend toward media convergence will accompany the rise of new media.

### Career perceptions

Among the core socialist values, dedication is one of the core elements at the individual level, which is the value evaluation of citizens’ professional code of conduct and fully reflects socialist professionalism. The more accurate the cognition of occupation, the more accurately one can position one’s role in society and form reasonable occupational values, instead of falling into self-denial, cynicism, blind arrogance, and other negative perceptions of occupational values. For instance, a student in the medical field, whose initials are represented by the code XYW, views quite brought immense of encouraging material in the short videos.

*On Tiktok, I saw first-hand medical professionals rescuing patients and tending to the wounded. They have my utmost respect. A video uploaded on Tiktok a few months ago in which Doctor Zhu helped an older gentleman quickly rise to prominence. I like this doctor's Tiktok posts, and I appreciate that she often shares motivational fitness videos. Arguments have been made that youngsters are being misled by short videos. Actually, in my opinion, it is conditional.* (A 20-year-old student describes the beneficial aspects of viewing Tiktok on a smartphone. The interview was conducted at one university in Guangxi, China, in June 2022.)

After the rise of social cognitive psychological research with contents such as self, perception, attitude, cognition and emotion, several methods to measure these psychological elements appeared, and many scholars created some measuring scales that are still in use today. Self-report scale is the most commonly used method in psychometric measurement. In this method, the authors design a series of questions about mental states or mental activities, and subjects answer these questions one by one according to their psychological feelings or past behaviours.

## Discussion

Students at modern universities are significant users of short video services (Luo et al., 2013; Guo et al., 2014; Olowo et al., 2020). They are enthusiastic about news information suggestions, the distribution of short videos, data visualization news, chatbots, and intelligent audio-visual applications (Andersson et al., 2018; Rajkumar and Ganapathy, 2020). Under the effect of the media circumstances generated by a short video, what is their value orientation as the cornerstones of the cause of socialism with Chinese characteristics in the present revolution? It has a significant impact on the future development of socialism with Chinese characteristics, which can be summarized as Chinese-style modernity (Zhu et al., 2021). Using it as the subject of study has commonplace and substantial practical applicability (Rouse, 2007).

Students at universities with greater media literacy education get more instruction in scientific perspectives on complicated information, as well as more education on media literacy (Hobbs and Jensen, 2009; Share et al., 2019). They would be more competent with critical media literacy in the areas of information screening, information analysis, information utilization, and other elements (Alvermann and Hagood, 2000). As a consequence, they are better able to cope with knowledge in a comprehensive manner (Coiro, 2021; Isnawati and Yusuf, 2021), which enables them to utilize information more successfully in the context of the interaction between themselves and information from the outside world. In the process of interacting with short videos, students depend on their experience in self-judgment, introspective thought, and observation of differences, among other areas. As a result, students have a better ability to comprehend how to get useful information from various media information platforms.

### Positive correlation variables of self-perceived level values metric: career perception, impression prediction, freedom of choice, thinking cost

According to the present research, the interview demonstrates that university students learn more knowledge about their prospective future careers using the short video platform. Following the findings of Lagu and Greysen (2011), the findings of the present study indicate that social media users gain from the development of professional ethics, professionalism, and responsibility. University students pay greater attention to their self-image and the expectations of others and incorporate more empathy into their information communication behaviour during short video interactions (Chretien and Kind, 2013). It is more conducive to establishing superior communication expectations. With the assistance of the information platforms and resources like TikTok, university students may overcome the current information barriers caused by algorithms at the level of self-awareness, which is favourable to the formation of positive values. As a consequence, university students are prepared to use more intellectual energy and mental energy in the process of acquiring short videos, which assists in their capacity to integrate their necessitous. Thus, youngsters are able to elaborate on videos depicting social public events with substantial societal impact, thus enhancing their self-perception values.

### Negatively correlated variables of the values metric: utility satisfaction, information realism, entertainment satisfaction

It can be observed from utility satisfaction that as utility satisfaction, information realism, and entertainment satisfaction grow, university students’ self-perceived values become more divergent, or the establishment of benign values becomes less favourable. On the one hand, university students’ utility satisfaction reflects the use of short videos for knowledge acquisition (Margaris et al., 2019). On the other hand, it indicates that people may have a dependency on short videos, such as being content with the algorithm suggestion process (Campana and Delmastro, 2017; Zhang T. et al., 2020). This obsessive dependence confines adolescents in a media environment centred on their interests, and they may also be addicted to virtual social interaction. Under the cultural ecology of short videos, university students are more susceptible to being influenced by unidirectional information. Some short videos of curiosity, for instance, might easily hinder the healthy development of an individual’s values. Information realism indicates, to some degree, an individual’s confidence in short videos (Kaur and Dubey, 2014). Due to the fact that the algorithm of short video platforms does not include a system for automatic detection, false news, clickbait news, and post-truth news occasionally occur (Kumar and Shah, 2018; Li and Cho, 2022). Individuals’ excessive reliance on the veracity of this news is likely to have a detrimental effect on their self-perceived social values.

Under the premise of human-computer interaction in short video communication, it is evident that self-perception values are strongly connected with career perception, impression prediction, freedom of choice, and thinking cost. Whereas utility satisfaction, information realism, and entertainment satisfaction are negatively correlated with values at the level of self-perception.

## Conclusion

According to the semi-structured interview and content analysis, the dissemination of short video values is based on the level of self-perception, with the following components: career perception, impression prediction, freedom of choice, thinking cost, utility satisfaction, information realism, and entertainment satisfaction. This paper analyses the values dissemination of short videos from the perspective of the self-perception of university students, which provides ideas for optimizing algorithms, enhancing the mode and behaviour of individual self-perception, and the beneficial guidance of external self-perception to individuals. This paper examines three aspects of self-perception, symbolic interaction, and social networks. Through experimental comparisons of the three types of perception, we find that self-perception influences people’s perceptions of others and their self-perception. On this basis, we can find that when interacting with others, symbolic interaction can enhance individuals’ perceptions of others as well as their self-perceptions.

Therefore, symbolic interaction is not only beneficial for social interaction, but also the psychological health and integrity of the individual. Symbolic interaction is a process in which multiple subjects participate in social life, in which each individual understands society through social practice, so that he or she can develop and improve oneself and make progress. Self-perception is one of the most important factors that help us to discover and define ourselves and to influence others. Symbolic interaction and social interaction are a process in which we both perceive ourselves and others and connect with them. In the process of symbolic interaction, we are constantly self-perceiving both ourselves and others; at the same time, we can perceive the symbolic meaning in others. From self-perception, we are able to enhance our self-worth and self-image. So, in this process, we are influenced by symbolic interaction and self-perception, which affects others and changes us. So, when symbolic interaction can change us, we need to actively perceive others and engage in self-expression and symbolic interaction. In turn, symbolic interactions can enhance an individual’s self-perception and also change their own perceptions and emotions. We can improve our ability to express ourselves, communicate and judge right and wrong, as well as our self-awareness and social adaptability in our interactions with others; and we can be more active in our interactions with others.

### Theoretical and managerial contributions

The contributions of this paper are manifestly significant from both theoretical and managerial standpoints. Firstly, it underscores the dual nature of symbolic engagement, demonstrating that it not only facilitates identification and connection with others but also actively promotes understanding and meaningful connections. Symbolic interaction enriches the process of deciphering symbolic meanings in interpersonal communication. Therefore, a profound understanding of how to actively discern and convey symbolic meanings is imperative. This theoretical insight enhances our comprehension of the dynamics of symbolic engagement.

Secondly, this paper extends the current discourse on social media governance concepts, emphasizing the importance of heightened awareness and cognitive abilities in effective symbolic communication. It highlights the role of symbolic engagement in bolstering self-perception, fostering interpersonal connections, and facilitating active participation in online interactions. From a managerial perspective, this insight underscores the need for a more nuanced approach to social media governance, considering the cognitive capacities required for effective symbolic communication.

Furthermore, from a managerial standpoint, it underscores the responsibility of social media regulatory bodies to promote self-awareness regarding symbolic engagement, enhance cognitive abilities, and facilitate self-discovery in the digital realm. This managerial implication underscores the role of regulatory bodies in shaping the online landscape to encourage responsible and meaningful symbolic interactions.

Lastly, this paper elucidates how optimizing self-perception among university students, including understanding the algorithmic mechanisms of short video dissemination, can contribute to the dissemination of values through such videos. From a managerial perspective, this insight highlights the potential for leveraging short videos to enhance university students’ professional cognition. Overall, the study provides valuable theoretical insights and practical guidance for both scholars and social media managers.

### Limitations and future studies

Our study is designed as an observational study, which means that it captures data at a specific point in time and explores associations between TikTok usage and self-perception among Chinese university students. While our research provides valuable insights into these associations, it is essential to acknowledge that establishing causality in observational studies can be complex. We recognize that further longitudinal and experimental research would be required to establish a causal relationship definitively, in which we have identified in the limitation section of our research ending section. Future studies could investigate changes in TikTok usage patterns and their impact on self-perception over an extended period. Additionally, experimental designs could be employed to manipulate TikTok exposure to assess its direct influence on self-perception. It is also recommended that additional research be conducted on the following concerns in the future. When university students are exposed to short videos, it is advised that they be guided to focus their efforts on forecasting their own and other’s perceptions and to think thoroughly about the content information. Additionally, it is vital to investigate other genres, formats, and platforms in order to diminish university students’ reliance on a particular algorithm application. The information authenticity screening feature was developed to help university students have a positive opinion of the legitimacy of knowledge. Future studies should also be placed on lowering the burden of entertainment information distribution.

## Data availability statement

The original contributions presented in the study are included in the article/supplementary material, further inquiries can be directed to the corresponding author.

## Ethics statement

Ethical approval was not required for the study involving human samples in accordance with the local legislation and institutional requirements. Written informed consent for participation in this study was provided by the participants’ legal guardians/next of kin. Written informed consent was obtained from the individual(s) for the publication of any potentially identifiable images or data included in this article.

## Author contributions

YM, GX, and SMS: conceptualization. JZ and GX: data curation, validation, and writing – review & editing. JZ: formal analysis, methodology, and supervision. JZ and HH: funding acquisition. YM and GX: investigation. YM and HH: project administration and resources. YM and SMS: software. JZ and SMS: visualization. YM: writing – original draft. SNS: writing – review and editing, resources, visualization, and investigation. All authors contributed to the article and approved the submitted version.
